# Clamping of the endotracheal tube to preserve the level of PEEP during disconnection from the ventilation circuit – a survey on routine practice

**DOI:** 10.1186/s12871-026-03849-1

**Published:** 2026-04-22

**Authors:** Christian Waydhas, Torben Brod, Bernhard Gliwitzky, Frank Herbstreit, Niklas Menzen, Sabrina Pelz

**Affiliations:** 1 Intensive Care Unit of the Department of Trauma, Hand and Reconstructive Surgery, University Hospital Essen, Hufelandstr. 55, 45137 Essen, Germany; 2 Department of Emergency Medicine, Hannover Medical School, Hannover, Germany; 3 MegaMed Emergency Management GbR, Maikammer, Germany; 4 Department of Anesthesiology and Intensive Care, University Hospital Essen, Essen, Germany; 5 Interdisziplinäre Notaufnahme, University Hospital Münster, Münster, Germany; 6 Department of Medicine, Intensive Care Unit, University Hospital Tübingen, Tübingen, Germany; 7 German Interdisciplinary Association of Critical Care and Emergency Medicine, Sektion Qualität und Ökonomie, Berlin, Germany; 8 German Interdisciplinary Association of Critical Care and Emergency Medicine, Sektion Strukturen Klinische Akut- und Notfallmedizin, Berlin, Germany; 9 German Interdisciplinary Association of Critical Care and Emergency Medicine, Sektion Notfall- und Katastrophenmedizin, Berlin, Germany; 10 German Interdisciplinary Association of Critical Care and Emergency Medicine, Sektion Pflegeforschung und Pflegequalität, Berlin, Germany

**Keywords:** Mechanical ventilation, Positive end-expiratory pressure, Lung volume, Decruitment, Respiratory distress syndrome, Ventilation circuit, Endotracheal tube

## Abstract

**Background:**

Clamping of the endotracheal tube during disconnection from the ventilatory circuit appears to be commonly used to prevent abrupt loss of positive end-expiratory pressure (PEEP) in invasively ventilated patients. Despite its physiological rationale, this maneuver is not addressed in major guidelines, and data on its prevalence of use and safety are lacking. *Objective*: To assess the prevalence of endotracheal tube clamping during ventilator disconnection, describe clinical situations in which it is applied and explore reported adverse events associated with its use.

**Methods:**

We conducted a cross-sectional, anonymized electronic survey among members of the German Interdisciplinary Association of Critical Care and Emergency Medicine (DIVI).

**Results:**

A total of 1,344 participants completed the survey. Overall, 87% reported using the clamping maneuver at least occasionally. In only 7.6% of cases, its use was based on local guidelines or standard operating procedures. Clamping was applied across different ventilation modes and with various types of clamps. A total of 11.4% of respondents reported having observed adverse events temporally associated with the maneuver, including airway device damage.

**Conclusions:**

Endotracheal tube clamping during ventilator disconnection appears to be a widespread but largely non-standardized clinical practice. Reported safety concerns underscore the need for systematic evaluation of its clinical benefits and risks, as well as for the development of evidence-based recommendations and standardized training concepts.

**Trial registration:**

The study was registered at the German Clinical Trials Register on November 3, 2025, registration number DRKS00038337 at https://www.drks.de/DRKS00038337.

**Supplementary Information:**

The online version contains supplementary material available at 10.1186/s12871-026-03849-1.

## Background

Positive end-expiratory pressure (PEEP) is a fundamental component of invasive mechanical ventilation [1, 2]. The level of PEEP is determined, apart from local ventilation strategies, by various factors such as the severity of the respiratory insufficiency, the level of F_i_O_2_ required for adequate oxygenation, and the underlying disease or condition [3, 4]. Higher PEEP levels have been shown to be beneficial for more severe adult respiratory distress syndrome (ARDS) [5, 6]. Among the main objectives of the application of PEEP are maintaining alveolar ventilation to enhance oxygenation, improving the perfusion-ventilation ratio, and minimizing ventilator-induced lung injury. Furthermore, the level of PEEP has a direct influence on cardiac function [7].

An acute drop in the level of PEEP has been demonstrated to induce alveolar decruitment and secondary lung damage in both animal models [8, 9] and human patients [10, 11]. The collapse appears to manifest within seconds after the cessation of PEEP [12]. The severity of the adverse effects seems to be correlated with the level of PEEP. Furthermore, repeated reductions in PEEP in a large animal model of hypervolemic individuals were associated with the development of pulmonary edema [13]. However, the clinical long-term relevance remains uncertain [14] and we are not aware of any study evaluating a benefit-risk assessment.

Common clinical situations during which PEEP drops to virtually zero include any disconnections of the endotracheal tube from the ventilatory circuit. This occurs when the ventilating machine is changed, such as during transfers of a ventilated patient between the prehospital rescue system, the emergency department, the intensive care unit, the operation room, other hospitals, and other situations. During the intensive care unit (ICU) stay regular disconnections occur when the heat and moisture exchanger (HME)-filters or active humidification devices are changed or when soiled or obstructed catheter mounts or ventilatory tubes are exchanged, among other occasions.

To mitigate the undesirable effects of a (sudden) drop of PEEP, a widely adopted practice in intensive care units involves clamping of the endotracheal tube during ventilation with higher levels of PEEP with a clamp whenever the endotracheal tube or tracheal cannula is disconnected from the ventilatory circuit. Despite its physiological rationale, the clinical relevance of this maneuver remains uncertain. Notably, the clamping maneuver itself may entail risks, including airway obstruction and tube or cuff damage. Data on how frequently this practice is used, under which clinical conditions it is applied, and which adverse events are encountered in routine clinical care are scarce. A literature search regarding the frequency of use yielded only a single short communication by McCormack on the practice in a New Zealand hospital [15].

Therefore, we conducted a cross-sectional survey among healthcare professionals working in intensive care units, emergency departments, and prehospital emergency services to assess the prevalence of endotracheal tube clamping during ventilator disconnection, describe clinical situations in which it is applied, and explore reported adverse events associated with its use.

## Methods

We conducted a cross-sectional, anonymized electronic survey following the CROSS-checklist [16].

The survey instrument was developed by the authors, an interprofessional team, comprising intensivists, emergency physicians, intensive care nurses, emergency care nurses, and paramedics. Owing to the absence of validated questionnaires or comparable studies addressing endotracheal tube clamping during ventilator disconnection, the survey items were formulated based on the clinical expertise and consensus within the study group. To enhance clarity, consistency, and face validity, the questionnaire was pilot-tested and subsequently revised. The pilot test was performed among coworkers of the authors so that intensivists and emergency physicians, intensive care nurses, emergency care nurses, and prehospital emergency personal were equally involved. In six questions the wording of predefined answers was clarified, in one item the sequence of predefined answers was changed, and for one answer a new option was introduced. The compulsory question, whether the participants had observed an adverse event during their professional experience required a yes-or-no answer. In the case of a positive answer the participants were asked to name such events in free text. Since this was exploratory, no pre-test for interpretability was performed. It was assumed that no incidence of adverse events could be deduced from this type of survey. The final survey consisted of 12 items and was designed to be completed within approximately five minutes. It is important to note that no data concerning individual patients were collected during the survey. The translated questionnaire is presented in Appendix 1 (electronic supplemental material). The survey was administered using SurveyMonkey® software, ensuring full anonymization and allowing only one response per invited participant. The survey was conducted between November 28 and December 14, 2025.

The survey was distributed electronically to all 5,126 members of the German Interdisciplinary Association of Critical Care and Emergency Medicine (DIVI), including physicians, nurses and respiratory therapists working in intensive care units or emergency departments, as well as prehospital emergency service personnel.

The anonymized raw data were exported to an Excel file (Microsoft® Excel for Mac, version 2019) and analyzed descriptively. The response rate was calculated as the number of completed surveys divided by the number of invited participants. All questions were compulsory, except for the free text inputs.

The study was approved by the Ethics Committee of the University of Duisburg-Essen on October 30, 2025 (registration number: 25-12751BO). The study adhered to the Declaration of Helsinki in its present form. Informed consent to participate was obtained from all participants by clicking on the agreement button. Only then could they proceed to the survey. Before leaving the survey, all participants could require a print-out of the participant information sheet, their consent, and their answers. The study was registered at the German Clinical Trials Register on November 3, 2025, at https://www.drks.de/DRKS00038337

## Results

A total of 1,344 completed questionnaires were analyzed, corresponding to an overall response rate of 26.2%. Response rates were comparable between physicians (24.1%, *n* = 747) and other healthcare professionals (29.7%, *n* = 597). The predominant working area of the participants was the intensive care unit (ICU) in 75.2%, while the prehospital rescue service, emergency department, and operation room were represented less frequently. Most participants (78%) reported more than ten years of professional experience.

**Fig. 1:**
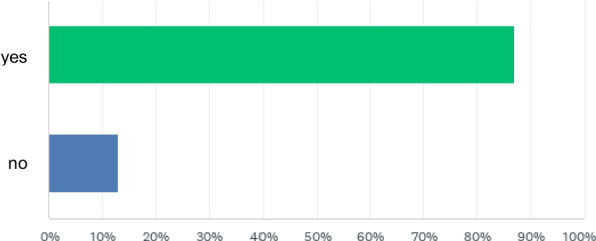
Is clamping of the tube to preserve PEEP during disconnection used, at least occasionally on your ward or in your work area, personally or by colleagues

Overall, 87% of respondents reported using endotracheal tube clamping to preserve PEEP during ventilator disconnection at least occasionally (Fig. 1). In contrast, 13% (*N* = 172) did not employ clamping to preserve PEEP. The rationales for eschewing clamping in order to preserve PEEP are summarized in Table 1.

**Table 1:** Reasons for not using clamping of the tube to preserve positive end-expiratory pressure during disconnections from the ventilator (*N* = 172, multiple answers were possible*)

Options	Percentage	Number
It is not common in our service	62.8	108
There is no scientific proof or guideline recommendation	50.8	86
There is no standard operating procedure for it	39.0	67
It is not considered necessary	33.1	57
Because the tube can be damaged	25.6	44
Because the risk is too high	11.6	20
We apply other measures to avoid a loss of PEEP	5.8	10
The superiors do not like it	5.2	9

^*^Due to the varying background of the participants (e.g. scientific vs. practical knowledge) an overlap in some of the options was allowed

Among those who utilize clamping, 7.6% indicated that its application was based on a standing order or a standard operating procedure. In most cases, clamping was reported to be performed either as an unwritten local practice (46.4%) or based on individual preference (46%). A majority of users (58.4%) reported a threshold for the level of PEEP of 10 cmH_2_O or more for initiating the maneuver. 17.2% of respondents use a level of PEEP of 6 cmH_2_O or more, while 6.6% would require a PEEP of at least 15 cmH_2_O. The remainder either applies it at any level of PEEP (4.5%) or based on individual preference (13.3%).

**Fig. 2:**
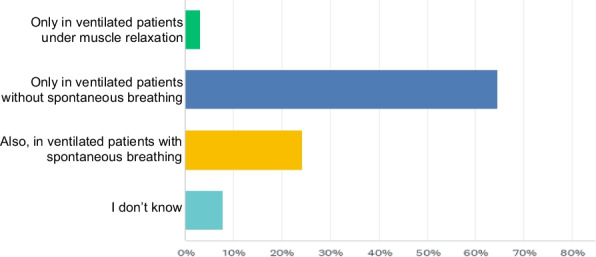
Under what ventilation conditions do you use the clamping of the tube

Figure 2 illustrates the reported breathing pattern of patients during the application of the clamping maneuver. The clamping maneuver was reported to be used not only in fully controlled ventilation but also in patients with spontaneous breathing activity by 24.3% of respondents. Most respondents reported using either a single metal hose clamp (59.6%) or a single plastic clamp (23.7%). Other options, such as an ECMO-clamp, two plastic or metal clamps with a 90-degree offset, were each utilized by less than 5% of users.

**Fig. 3:**
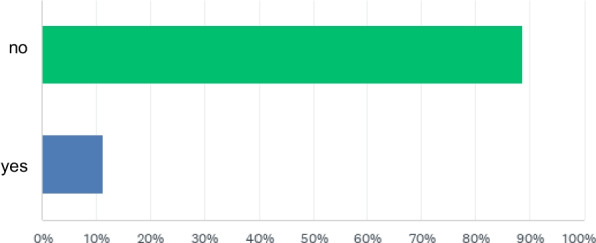
Have you observed adverse events from the clamping

Adverse events temporally associated with the clamping maneuver were reported by 11.4% of the participants (Fig. 3). A total of 137 individual adverse events were described, with multiple events potentially reported by a single participant. The most frequently reported adverse events (*N* = 104) involved technical device-related problems such as damage to the endotracheal tube or the cuff inflation line (*n* = 56). Additional reported events included accidental clamping of armored endotracheal tubes (*n* = 26) and difficulties in releasing metal clamps (*n* = 22). Further reported adverse patient reactions included coughing and pressing (*N* = 13), or anxiety and agitation (*N* = 10). Respondents indicated that several of these adverse events were accompanied by clinically relevant consequences including oxygen desaturation (*N* = 7), arterial hypotension (*N* = 2), bronchospasm (*N* = 2), pneumothorax (*N* = 1) or even cardiac arrest (*N* = 1). In several cases, an emergency exchange of the endotracheal tube was reported to have been required.

## Discussion

In this cross-sectional survey among healthcare professionals working in intensive care, emergency, and prehospital settings, we identified three novel findings. First, clamping of the endotracheal tube to preserve PEEP is a widespread practice. Second, its application is rarely guided by formal recommendations or standard operating procedures and instead predominantly relies on unwritten local practices or individual clinical judgment. Third, a relevant proportion of respondents reported adverse events temporally associated with the maneuver, indicating potential safety concerns.

Approximately 90% of respondents indicated that the practice of clamping the tube to preserve PEEP during disconnection from the ventilator was employed, at least occasionally, on their ward or in their work area, either personally or by their colleagues. However, published data on the frequency of this practice are limited. A case report from a New Zealand hospital revealed that the practice was not prevalent in the United Kingdom at the time (2010) [15]. Our findings from Germany provide a strong contrast to this observation. The growing interest in scientific studies investigating the preservation of PEEP by clamping in various countries, including Canada, France, Sweden, Switzerland, the Czech Republic, the United States, and others, suggests that this maneuver may be commonly employed in other regions worldwide [8–13, 17, 18].

The purpose of clamping the endotracheal tube is to maintain a constant level of PEEP during the disconnection process. This practice assumes that PEEP is beneficial and that even a brief reduction of PEEP during disconnection would be harmful. An adverse effect on alveolar recruitment by a complete drop of PEEP has been demonstrated both in animal models and human patients [8–12]. In ventilated patients with acute lung injury or ARDS a decruitment of 373 ml ± 250 ml was observed after disconnection from the ventilator with a drop of PEEP from 15 to 0 cmH_2_O [11]. A potentially beneficial effect would be particularly anticipated in patients undergoing ventilation with higher levels of PEEP. However, which exact level of PEEP this might be, is elusive. In our study, most participants utilized a threshold of a PEEP of 10 cmH_2_O or more to perform the maneuver.

Contrary to the anticipated adverse effects of a decrease in PEEP, some evidence suggests that these negative consequences may be mitigated by a subsequent recruitment maneuver. Furthermore, several experimental studies have demonstrated that re-recruitment following the restoration of PEEP occurs at the same rate as the initial decruitment. (literature in [19]). Additionally, a decrease in PEEP may be frequently observed during suctioning procedures that cannot be mitigated by clamping [20, 21].

Despite potential beneficial physiological effects of PEEP preservation and observed adverse short-term effects in animal models and humans following a sudden decrease in PEEP, their impact on patient outcomes remains indeterminate. To our knowledge, there is no evidence demonstrating favorable effects on ventilator time, mortality, or other outcome parameters. Studies assessing patient-centered outcomes are needed to clarify the clinical benefit-risk balance of the clamping maneuver. Recommendations regarding the clamping maneuver are not included in any major guideline. The absence of guideline recommendations or standard operating procedures was one of the primary reasons for the non-use of the maneuver. This aligns with a predominantly unregulated use (either on individual preferences or through unwritten practice, collectively accounting for 92%) by those participants who employ clamping.

Only recently, it has been demonstrated that the type of clamp employed for compressing the endotracheal tube significantly influences the decrease in airway pressure during clamping [17, 18]. Although the ECMO-clamp demonstrated the most effective PEEP-preserving effect in these studies, it was utilized by less than 5% of the participants. Consequently, it is reasonable to infer that the potential PEEP-preserving effect of other types of clamps will be diminished in a significant majority of patients. Before using the clamping maneuver, clear recommendations and standards are required with respect to the technical aspects of its execution.

The high rate of adverse events has not been described yet and is a cause for concern. One in ten participants reported encountering adverse events at some time during the use of the clamping maneuver. It must be noted that this number does not allow for the calculation of an incidence (e.g., adverse events per 100 applications). It reflects observations of the participants during their professional experience, independent from the frequency of the application of the clamping maneuver. Nevertheless, the severity of the consequences that have been reported by the participants in conjunction with clamping maneuvers, such as permanent tube obstruction, (emergency) reintubation, pneumothorax, hypoxia, hypotension, and cardiac arrest, warrants a closer analysis. Unfortunately, we cannot construct a sequence of events from the initial technical device-related problem or the patient reaction neither to a consecutive physiological response nor to necessary urgent or emergency interventions or to the severity of the event. The most common problem was damage to the tube or the cuff inflation line. This may be a consequence of the type of clamp that was used. We cannot attribute the damage to a specific type of clamp, but several respondents indicated that they switched from a metal to a plastic clamp thereafter. Some users applied a pad between the clamp and the endotracheal tube to avoid direct contact of the metal with the tube. Both solutions raise the question whether the level of PEEP will then be maintained as intended. The problem of releasing the metal clamp also highlights the technical challenges associated with a non-standardized maneuver. It remains unknown whether and how often this was a major problem (with physiological consequences, e.g. desaturation) or not. Clamping of an armored tube appears to be a significant but preventable error. This results in a permanent partial or (sub)total occlusion of the tube. For such patients it may be assumed that an unplanned change of the endotracheal tube was necessary, but we do not know how often an emergency intervention was required or the situation remained controllable. We cannot clearly elucidate the reasons behind its frequent occurrence. One possible explanation could be the lack of adequate job training combined with a lack of guidelines and standing operating procedures, as reported by most of the respondents. Another problem appears to be clamping in patients with spontaneous breathing efforts. In addition to anxiety, agitation, and stress, the development of low-pressure barotrauma appears to be an imminent risk. We cannot comment on the frequency of these events as we are not able to relate the use of the camping maneuver specifically in patients with spontaneous breathing. To avoid this anticipated risk, it has been recommended that clamping of the tube should be avoided in patients with spontaneous breathing as there may be a risk of negative pressure pulmonary edema. [22]. In future studies it will be necessary to calculate an incidence of adverse events and provide a sequence of events from the initial problem (e.g. damaged tube or cuff line, stenosis or obstruction of armored tube, difficulty to release the clamp, coughing) to consecutive physiologic disturbances, and required emergency or urgent interventions, as well as a grading of the severity of such events. Until such data will be available the clamping of an endotracheal tube to preserve PEEP should be considered as a potentially dangerous maneuver, requiring thorough education of the personal involved and the institution of local standard operating procedures. Recently, several devices have been proposed to facilitate the clamping procedure [23–25] but data on their practicability and a comprehensive risk–benefit assessment are lacking.

The study has several limitations. Some interesting survey questions might be missing. However, to our knowledge, no data from previous studies are available. Consequently, no references could be utilized to develop the survey questions. The authors are specialists in prehospital rescue, emergency, and intensive care in their respective professions. Furthermore, the pilot trial provided feedback that facilitated the adaptation of the survey.

Another potential limitation is the selection bias of the participants. However, this group is representative of medical and other healthcare professionals involved in prehospital, emergency, and intensive care. Professionals who are not members of the DIVI may have provided an even broader overview, but there are no evident indicators that this would lead to significantly different results. The response rate was only moderate and may have biased the results. However, if none of the non-respondents used the clamping maneuver, still close to one quarter of all invited participants would apply it. An alternative would have been to use a comprehensive database containing all German intensive care units and emergency departments. In such a scenario, the relevant directors would have been contacted. However, our primary objective was not to inquire about departmental policies, but rather to ascertain whether individuals from various professions and work environments indeed carry out the procedure. Lastly, the participants were from Germany, Austria and Switzerland. The practice of clamping, the technical knowledge, and the education might differ in other countries. We cannot comment on this, because we are not aware of any such data from elsewhere. However, the growing interest in the physiological effects of the maneuver, as reflected by many recent studies in many other countries suggests its general relevance.

## Conclusions

Our study presents three key findings: (1) Clamping of the endotracheal tube to preserve the level of PEEP during disconnection from the ventilation circuit is a common clinical procedure in intensive and emergency care settings; (2) The rationale for this procedure (clamping maneuver) is rarely included in standing orders, instead relying on unwritten practice or individual preference; and (3) a relevant proportion of respondents reported serious adverse effects temporarily associated with the maneuver. While there is a growing interest in evaluating the benefits of the clamping, further research is needed to clarify its impact on clinical outcomes, establish patient selection criteria, develop safe technical solutions, create evidence-based recommendations, and design educational programs.

## Supplementary Information

Supplementary Material 1: Includes a translation from the original German survey in English language.

## Data Availability

The datasets analyzed during the current study are not publicly available due to planned further analysis but are available from the corresponding author on reasonable request, earliest 12 months after publication of the accepted manuscript.

## Abbreviations

ARDS: Adult respiratory distress syndrome
DIVI: German Interdisciplinary Association of Critical Care and Emergency Medicine
F_i_O_2_: Fraction of inspired oxygen
ICU: Intensive care unit
PEEP: Positive end-expiratory pressure
