# Supplementary material for: Clamping of the endotracheal tube to preserve the level of PEEP during disconnection from the ventilation circuit – a survey on routine practice
Source: BMC Anesthesiol. 2026 Apr 22;26:253. doi: 10.1186/s12871-026-03849-1 (PMC13101170; doi:10.1186/s12871-026-03849-1)
Supplement: Supplementary file 1 — Supplementary Material 1: Includes a translation from the original German survey in English language. [file 12871_2026_3849_MOESM1_ESM.docx]

## Supplementary Material 1 - Questionaire

**Clamping of the endotracheal tube to preserve the level of PEEP during disconnection from the ventilation circuit – a survey on routine practice**

Christian Waydhas^1,7^, Torben Brod^2,8^, Bernhard Gliwitzky^3,9^, Frank Herbstreit^4^, Niklas Menzen^5^, Sabrina Pelz^6,10^

**Appendix 1 - Questionnaire**

**Question 1**

In which field are you currently mainly active?

- Rescue service
- Emergency room
- Intensive care unit
- Operating room

**Question 2**

Which professional group do you belong to?

- Rescue personnel
- Nursing staff
- Medical staff
- Respiratory therapy staff
- Other

**Question 3**

How many years of professional experience do you have?

- In training
- < 5 years
- 5 – 10 years
- > 10 years

**Question 4**

Is clamping of the tube to preserve PEEP during disconnection used, at least occasionally, on your ward or in your work area (by you personally or by colleagues)?

- Yes
- No

If Yes: continue with question 5

If No: continue with question 4A

**Question 4A:**
Why not? Multiple answers are possible.

- The superiors do not want it
- It is not common in our service
- There is no standard operating procedure for it
- It is not considered necessary
- There is no scientific proof or guideline recommendation
- Because the tube can be damaged
- Because the risk is too high? (please specify what risks)
- We apply other measures to avoid the loss of PEEP (please specify)

**Question 4B**:
What is your opinion? Do you think it is helpful to clamp the tube to preserve the PEEP?

- Yes, I personally think that clamping the tube is helpful
- No, I personally don't think that clamping the tube is helpful
- No opinion

*Continue to Conclusion (Submit)*

**Question 5**

How often is this measure performed (clamping the tube to preserve the PEEP)?

- Occasionally, due to individual assessment
- Regularly, there is an unwritten routine in my field of work
- Regularly, there is a standing order or standard operating procedure

**Question 6**

During which of the following occasions, if indicated, do you perform the clamping? Multiple answers are possible.

- During emergency missions (prehospital)
- As part of (secondary) transfer transports (to or from the hospital)
- As part of transfers within the hospital (intrahospital)
- When changing the ventilator (within my work area)
- When changing HME filters, active humidification, or catheter mount
- When changing the ventilation tube system

**Question 7**

Is there a specific level of PEEP above which the clamping is carried out? Please choose the most appropriate answer.

- At every PEEP above 0 cmH_2_O
- at least 6 cmH_2_O or higher
- at least 10 cmH_2_O or higher
- at least 15 cmH_2_O or higher
- at least 20 cmH_2_O or higher
- According to individual assessment
- I don't know

**Question 8**

Under what ventilation conditions do you use the clamping of the tube?

- Only in ventilated patients under muscle relaxation
- Only in ventilated patients without spontaneous breathing
- Also, in ventilated patients with spontaneous breathing
- I don't know

**Question 9**

Do you only use the clamping for specific clinical conditions?

- We use clamping regardless of specific clinical conditions
- We only use clamping for specific clinical conditions
- I don't know

**Question 9A** (if question 9 was answered with "for specific clinical conditions ")
For which specific clinical conditions do you use the clamping? Multiple answers are possible.

- Acute respiratory distress syndrome
- Chronic obstructive pulmonary disease
- Pulmonary contusion
- Pneumonia (general)
- Respiratory infections with highly contagious pathogens (e.g. COVID, influenza, TB)
- Other (Please specify)

**Question 10**

What type of clamp or clamping technique do you predominantly use?

- A plastic clamp
- Two plastic clamps, offset 90° from each other
- One metal hose clamp
- Two metal hose clamps, offset 90° from each other
- ECMO clamp
- Other (Please specify)

**Question 11**

Have you already observed complications from the clamping?

- No
- Yes (Please specify)

Completion and submission:
Thank you very much for your participation in the survey. We are very excited to see the results. We plan to publish them at a congress and in a medical journal.
PLEASE COMPLETE THE SURVEY by pressing the "COMPLETE SURVEY" button.
